# Ketamine Use for Successful Resolution of Post-ERCP Acute Pancreatitis Abdominal Pain

**DOI:** 10.1155/2017/7845358

**Published:** 2017-06-20

**Authors:** Suneel M. Agerwala, Divya Sundarapandiyan, Garret Weber

**Affiliations:** New York Medical College, Valhalla, NY, USA

## Abstract

We report a case in which a patient with intractable pain secondary to post-endoscopic retrograde cholangiopancreatography (ERCP) acute pancreatitis is successfully treated with a subanesthetic ketamine infusion. Shortly after ERCP, the patient reported severe stabbing epigastric pain. She exhibited voluntary guarding and tenderness without distension. Amylase and lipase levels were elevated. Pain persisted for hours despite hydromorphone PCA, hydromorphone boluses, fentanyl boluses, and postprocedure anxiolytics. Pain management was consulted and a ketamine infusion was trialed, leading to a dramatic reduction in pain. This case suggests that ketamine may be a promising option in treating intractable pain associated with ERCP acute pancreatitis.

## 1. Introduction

While subanesthetic ketamine infusion has been used to manage postoperative pain and opioid resistant pain, as well as acute pain and procedural sedation in the emergency department and in the pediatric population, there has been no study examining its use in ERCP pancreatitis in the adult population. We present a novel case in which intractable post-ERCP acute pancreatitis pain in an adult patient was successfully managed using a subanesthetic dose of ketamine infusion.

## 2. Case Description

The pain management service was consulted for a 24-year-old female with past medical history notable for polycystic ovarian syndrome. She presented with intractable abdominal pain, status post-endoscopic retrograde cholangiopancreatography (ERCP), for suspected choledocholithiasis/postcholecystectomy syndrome. Six weeks earlier, the patient underwent elective cholecystectomy and was discharged home uneventfully, with pain well controlled on oral analgesics. On the day prior to admission, the patient awoke with severe abdominal pain and presented to the emergency department. Her pain was reported as 10/10 on the numerical rating scale in severity and was described as sharp, located in her mid-abdomen above the umbilicus, radiating to the right upper quadrant and back, and accompanied by nausea. She was then admitted as an inpatient for further workup.

On hospital day #1 her pain was treated with morphine sulfate IV 3 mg q4h prn. She received four doses. On hospital day #2 the patient complained of persistent pain and her pain regimen was switched to Hydromorphone IV 0.2 mg q4h PRN. She was given 2 doses on hospital day #2. Abdominal imaging, which included right upper quadrant ultrasound and abdominal CT, revealed intrahepatic and extrahepatic bile duct dilatation, which was suspicious for possible biliary stones. Amylase and lipase were within normal limits. The primary team decided to pursue ERCP given the inconclusive workup.

Prior to the ERCP, the patient was given Hydromorphone IV 0.5 mg. She then underwent general anesthesia with endotracheal tube, with an uneventful anesthetic course. Anesthesia was induced with propofol and lidocaine and maintained with propofol boluses. She was also given a total of 100 mcg of fentanyl. Her ERCP showed diffuse common bile duct dilation of 12 mm and a possible amorphous filling defect in the common bile duct. The common bile duct was ballooned, a sphincterotomy was performed, and the pancreatic duct was stented. She was then extubated and brought to the PACU.

In the PACU, the patient complained of severe, excruciating, stabbing epigastric pain and demonstrated voluntary guarding and tenderness without distension. Amylase and lipase levels were elevated to 199 and 121, respectively, and her pain was attributed to post-ERCP acute pancreatitis. The anesthesia service immediately ordered Hydromorphone IV 2 mg, fentanyl 100 mcg, and midazolam 2 mg. With her pain still uncontrolled, approximately 1 hour later, while still in the PACU, the patient was given over the next hour one additional dose each of midazolam 2 mg, fentanyl 100 mcg, and hydromorphone 2 mg. Hydromorphone PCA with demand dosing 0.2 mg every 10 minutes was then started. The patient's pain remained uncontrolled over the next three hours, so an additional nurse-administered hydromorphone bolus was given. The pain management service was consulted at this point.

Given the patient's persistent pain despite hydromorphone PCA, hydromorphone boluses, fentanyl boluses, and postprocedural anxiolytics, a decision was made to start ketamine infusion (3 mcg/kg/min) at low dose for analgesia. The patient then experienced significantly improved pain which allowed her to leave the PACU stably and comfortably. This was accompanied by a reduction in PCA use overnight requiring only one demand dose and no additional nurse-administered boluses. The next morning she expressed that the pain had resolved to a feeling of mild soreness. On hospital day #4, the ketamine drip was discontinued and pain was controlled to a minimum. On hospital day #5, her labs had normalized, she was tolerating diet, and was discharged home.

## 3. Discussion

Current management for pain secondary to acute pancreatitis includes NSAIDs, acetaminophen, and opioids [1]. We present a case where traditional management for this type of pain was insufficient for analgesia and where a subanesthetic ketamine infusion was used to successfully alleviate pain.

A recent cohort study in pediatric pain management examined the use of low-dose ketamine infusion in the treatment of acute pain for multiple different diagnoses. The study showed reduction in pain scores after ketamine infusion for a pooled “inflammatory diseases” group that included patients with Crohn's disease and pancreatitis, in general, but did not delineate between the two diseases [2]. To our knowledge, our case study is the first report of ketamine used as an analgesic to treat intractable post-ERCP, specific, pancreatitis pain in the adult population.

The incidence of post-ERCP pancreatitis (PEP) is estimated to be 3.47%. Risk factors include female gender, young age, sphincter of Oddi dysfunction, absence of chronic pancreatitis, and history of prior PEP. Prevention in high-risk patients includes pharmacologic prophylaxis using anti-inflammatory medication and improved endoscopic techniques, such as stenting and faster biliary cannulation time [3].

In order to diagnose acute pancreatitis, two of the main three features must be confirmed. These features are abdominal pain typical for acute pancreatitis, serum amylase/lipase greater than 3 times the upper normal limit, and evidence of acute pancreatitis on CT scan. Initial assessment of severity is important for planning proper management. Mild acute pancreatitis is defined by lack of organ failure, moderate severity includes transient (<48 hr) organ failure, and severe is characterized by persistent (>48 hr) organ failure [4].

Analgesia does not modify the course or outcome of acute pancreatitis but supports patient comfort and patient-reported outcomes. Opioids, commonly used to treat pain in acute pancreatitis, work on the mu receptor pathway, both peripherally and centrally, to modulate the nociceptive response and perception of pain [5].

Classically, ketamine has been used for procedural sedation and postoperative pain. When given at anesthetic doses ketamine causes analgesia, amnesia, and sedation while having relatively little effect on respiration and overall hemodynamics [6]. At subanesthetic doses, the drug has been found to be effective in achieving analgesia in opioid resistant patients [7].

Ketamine's therapeutic effects have long been attributed to its ability to reversibly block the activity of N-methyl-D-aspartate (NMDA) receptors (NR). NR hyperactivity leads to sensitization to noxious stimuli and desensitization to opioids. Thus, blocking this pathway has dramatic effects on analgesia and opioid unresponsiveness. In chronic pain states, central and peripheral sensitization of pain pathways can cause the experience of pain to decouple from an appropriate stimulus. NRs are implicated in the plasticity that leads to sensitization, and blocking this plasticity with ketamine may help in chronic pain states. In the same way opioid receptors may be attenuated through NR-dependent mechanisms, leading to opioid tolerance. Blockade of this pathway may prevent, reduce, or delay desensitization to opioids [8, 9].

There are other pathways that may contribute to the analgesic effects of ketamine. An increase in substance *P* receptors has been implicated in greater pain sensitivity and loss of substance *P* receptors in less pain sensitivity. Ketamine is known to have a direct inhibitory effect on substance *P* receptors. There is some indication that ketamine may reduce the release of substance *P* into the synapse. Dopaminergic pathways may contribute as well. Ketamine has been shown to increase dopamine signaling, and preliminary evidence has shown that this is involved in pain reduction. Muscarinic acetylcholine receptor agonists may increase pain sensitivity thresholds, and ketamine may have some direct regulatory effects on those receptors. Serotonergic pathways may have pronociceptive roles, and ketamine may have an additional role in modulation [9].

Ketamine's analgesic effects were shown to be beneficial in many settings. Using ketamine in conjunction with morphine was shown to be superior for pain management in the postoperative setting, compared to morphine alone [7]. Ketamine has also been used in the emergency department for acute pain, where it showed similar analgesic effects to morphine [10]. A recent randomized, double-blind, placebo-controlled trial showed that after a subanesthetic infusion of ketamine over 15 minutes, patients reported significant decrease in pain scores for the first 30 minutes when compared to placebo infusions [11]. Another study showed increased analgesia of ketamine infusion when compared to placebo over a 2-hour period [12].

One shortcoming is the monitoring necessary to treat a patient with a subanesthetic ketamine drip. At our institution, the patient was required to stay in the PACU for 24 hours because of state regulations that require a higher level of monitoring for patients with a ketamine drip [13]. Additionally, she was transferred from the PACU to a higher acuity step-down unit before being transferred to a lower acuity floor. These rules may vary by state, but the additional resources for monitoring may be prohibitive for some institutions.

In summary, while ketamine has been used to manage postoperative pain, opioid resistant pain, and acute pain in the emergency department, evidence for its use to treat pain associated with an acute pancreatitis is limited. In our patient with severe post-ERCP pain associated with acute pancreatitis, opioids and coanalgesics were unable to control her pain. The addition of a subanesthetic ketamine drip offered quick and long-lasting analgesia. This could have been due to the ability of ketamine to modify the effects of opioids or the direct effect of ketamine itself on the nociceptive system. The patient was able to comfortably recover from her acute event, being discharged home with no complications. Further studies on the safety and the appropriate level of monitoring at subanesthetic dosing should be investigated to allow for further use outside of a highly monitored unit.

## References

[1] Gülen B., Dur A., Serinken M., Karcioğlu Ö., Sönmez E. (2016). Pain treatment in patients with acute pancreatitis: A randomized controlled trial. *Turkish Journal of Gastroenterology*.

[2] Sheehy K. A., Lippold C., Rice A. L., Nobrega R., Finkel J. C., Quezado Z. M. (2017). Subanesthetic ketamine for pain management in hospitalized children, adolescents, and young adults: a single-center cohort study. *Journal of Pain Research*.

[3] Szary N. M., Al-Kawas F. H. (2013). Complications of endoscopic retrograde cholangiopancreatography: How to avoid and manage them. *Gastroenterology and Hepatology*.

[4] Dupuis C. S., Baptista V., Whalen G. (2013). Diagnosis and management of acute pancreatitis and its complications. *Gastrointestinal Intervention*.

[5] Basurto Ona X., Rigau Comas D., Urrútia G. (2013). Opioids for acute pancreatitis pain. *The Cochrane database of systematic reviews*.

[6] Strayer R. J., Nelson L. S. (2008). Adverse events associated with ketamine for procedural sedation in adults. *The American journal of emergency medicine*.

[7] Weinbroum A. A. (2013). A single small dose of postoperative ketamine provides rapid and sustained improvement in morphine analgesia in the presence of morphine-resistant pain. *Anesthesia and Analgesia*.

[8] Lee S. K. (2012). The use of ketamine for perioperative pain management. *Korean Journal of Anesthesiology*.

[9] Iacobucci G. J., Visnjevac O., Pourafkari L., Nader N. D. (2017). Ketamine: an update on cellular and subcellular mechanisms with implications for clinical practice. *Pain Physician*.

[10] Barrett T. W., Schriger D. L. (2015). Move over morphine: is ketamine an effective and safe alternative for treating acute pain? journal club. *Annals of emergency medicine*.

[11] Sin B., Tatunchak T., Paryavi M. (2017). The use of ketamine for acute treatment of pain: a randomized, double-blind, placebo-controlled trial. *The Journal of Emergency Medicine*.

[12] Beaudoin F. L., Lin C., Guan W., Merchant R. C. (2014). Low-dose Ketamine Improves Pain Relief in Patients Receiving Intravenous Opioids for Acute Pain in the Emergency Department: Results of a Randomized, Double-blind, Clinical Trial. *Academic Emergency Medicine*.

[13] Zittel B. Practice Information: IV Drug Administration of Ketamine for the Treatment of Intractable Pain. http://www.op.nysed.gov/prof/nurse/nurse-iv-ketamine.htm.

